# Estimating the Capacity for ART Provision in Tanzania with the Use of Data on Staff Productivity and Patient Losses

**DOI:** 10.1371/journal.pone.0005294

**Published:** 2009-04-17

**Authors:** Stefan Hanson, Anna Thorson, Hans Rosling, Claes Örtendahl, Claudia Hanson, Japhet Killewo, Anna Mia Ekström

**Affiliations:** 1 Division of International Health (IHCAR), Karolinska Institutet, Stockholm, Sweden; 2 Division of International Health (IHCAR), Department of Public Health Sciences, Karolinska Institutet, Stockholm, Sweden; 3 Institute of Tropical Medicine, Antwerpen, Belgium; 4 Muhimbili University College of Health Sciences, Dar es Salaam, Tanzania; Canadian Agency for Drugs and Technologies in Health, Canada

## Abstract

**Background:**

International targets for access to antiretroviral therapy (ART) have over-estimated the capacity of health systems in low-income countries in Sub-Saharan Africa. The WHO target for number on treatment by end 2005 for Tanzania was 10 times higher than actually achieved. The target of the national Care and Treatment Plan (CTP) was also not reached. We aimed at estimating the capacity for ART provision and created five scenarios for ART production given existing resource limitations.

**Methods:**

A situation analysis including scrutiny of staff factors, such as available data on staff and patient factors including access to ART and patient losses, made us conclude that the lack of clinical staff is the main *limiting factor* for ART scale-up, assuming that sufficient drugs and supplies are provided by donors. We created a simple formula to estimate the number of patients on ART based on availability and productivity of clinical staff, time needed to initiate vs maintain a patient on ART and patient losses using five different scenarios with varying levels of these parameters.

**Findings:**

Our scenario assuming medium productivity (40% higher than that observed in 2002) and medium loss of patients (20% in addition to 15% first-year mortality) coincides with the actual reported number of patients *initiated on ART* up to 2008, but is considerably below the national CTP target of 90% coverage for 2009, corresponding to 420,000 on ART and 710,000 life-years saved (LY's). Our analysis suggests that a coverage of 40% or 175,000 on treatment and 350,000 LY's saved is more achievable.

**Conclusion:**

A comparison of our scenario estimations and actual output 2006–2008 indicates that a simple user-friendly dynamic model can estimate the capacity for ART scale-up in resource-poor settings based on identification of a limiting staff factor and information on availability of this staff and patient losses. Thus, it is possible to set more achievable targets.

## Introduction

Despite considerable international funding for antiretroviral treatment (ART) in heavily HIV-affected low-income countries in sub-Saharan Africa [1] scaling-up access to ART has been much slower than expected. The reasons are many and may include the effect of stigma on patient demand for ART, but is maybe first and foremost an overestimation of the output capacity of weak health systems. In its “3 by 5” target for Tanzania, the World Health Organization (WHO) initially aimed at 220,000 patients on ART by the end of 2005. This was 10 times higher than the number actually achieved [2]. Moreover, the fact that patients in sub-Saharan Africa often seek care at a late stage of the disease [3], causing a first–year AIDS mortality rate on ART of up to 15%, was not considered [4], [5], [6]. Increased emphasis on accountability makes it necessary for planners and donors to outline more implementable and achievable plans [7].

At the time the model was created Tanzania had an estimated adult HIV prevalence of 6.5%, corresponding to 1.4 (uncertainty range 1.2–1.5) million infected individuals [8]. The incidence of HIV has stabilised, and assuming a similar incidence over the last decade, approximately 175,000 people were estimated to be infected annually and 160,000 new individuals estimated to need ART every year in order not to die from AIDS [9], [10]. The Government of Tanzania started ART scale-up in mid-2004 in accordance with the five-year national Care and Treatment Plan (CTP) [11] developed together with the Clinton Foundation [12], mainly financed by the Global Fund (GFATM) [13] and President Bush's Emergency Plan for AIDS Relief (PEPFAR). The aim of the CTP is to provide ART for “as many HIV-infected residents as possible”, with 420,000 on ART by mid-2009. However, by February 2008, only 143,000 had been initiated on treatment (pc NACP).

**Figure 1:**
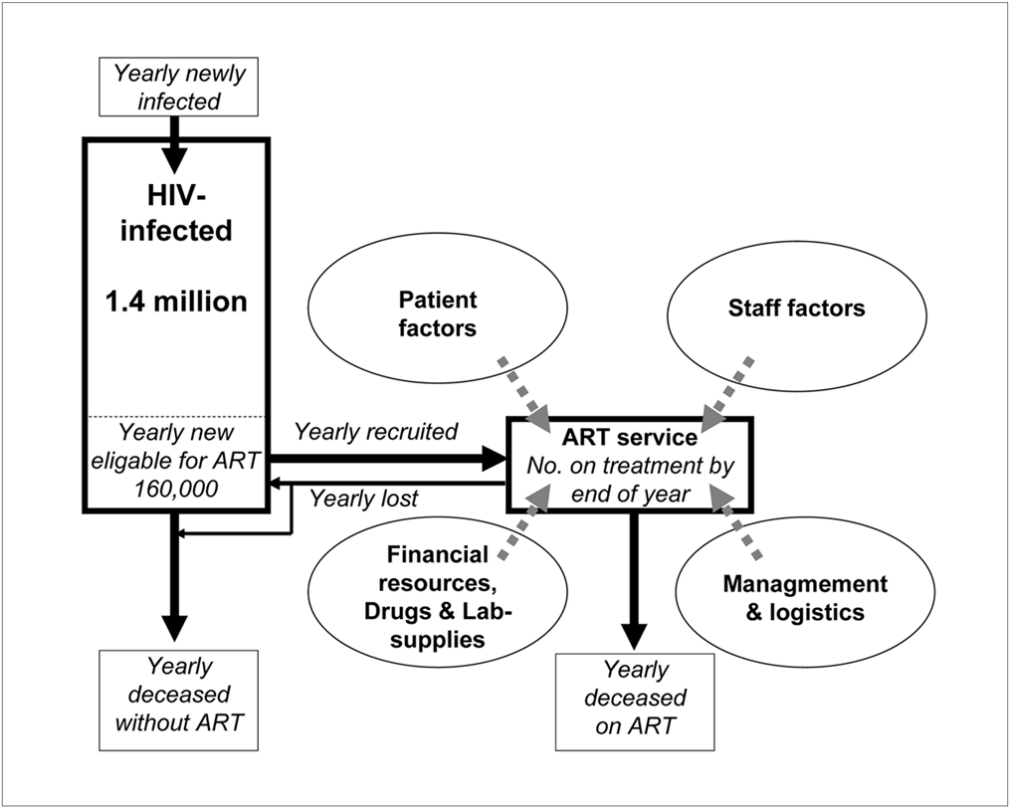
Conceptual model for ART provision. Conceptual framework with the flow of patients and the four major determinants of ART output in Tanzania.

To show that there are limitations to achieve these targets in weak health systems, and to demonstrate that it is possible for local planners in low income countries, to set more achievable targets, we worked out a simple method to estimate the maximum possible number of patients that could access ART in Tanzania. Estimates were based on the number of available clinical staff taking into account their productivity and consultation time needed to initiate vs maintain a patient on ART as well as the expected patient losses. We outline five different scenarios with varying levels of staff productivity and patient loss using observational data from human resource monitoring and available data on consultation time and patient losses derived from on-going ART programs. For a simplified model, we assumed that drug procurement and management issues would not be limiting factors during the implementation period (figure 1). The results therefore show the number of HIV-positive individuals in Tanzania that maximally could access ART under each scenario.

## Materials and Methods

We first reviewed the national Care and Treatment plan (CTP) for 2004–2009 including the ART output targets and the number of new Care and Treatment Centres (CTCs) that are to be accredited for scaling-up of access to ART.

We then made a situation analysis of the major bottlenecks for rapid scale-up of access to ART in Tanzania and other low-income countries including a review of the existing literature, unpublished documents and verbal information from key informants. These findings were summarized in a conceptual framework displaying the main determinants of ART output (figure 1).

We proceeded to create a formula containing the main factors for ART output i.e. the number on treatment by the end of each year, including the number of new and existing patients, the availability and productivity of clinical staff as well as patient losses.

**Figure 2:**
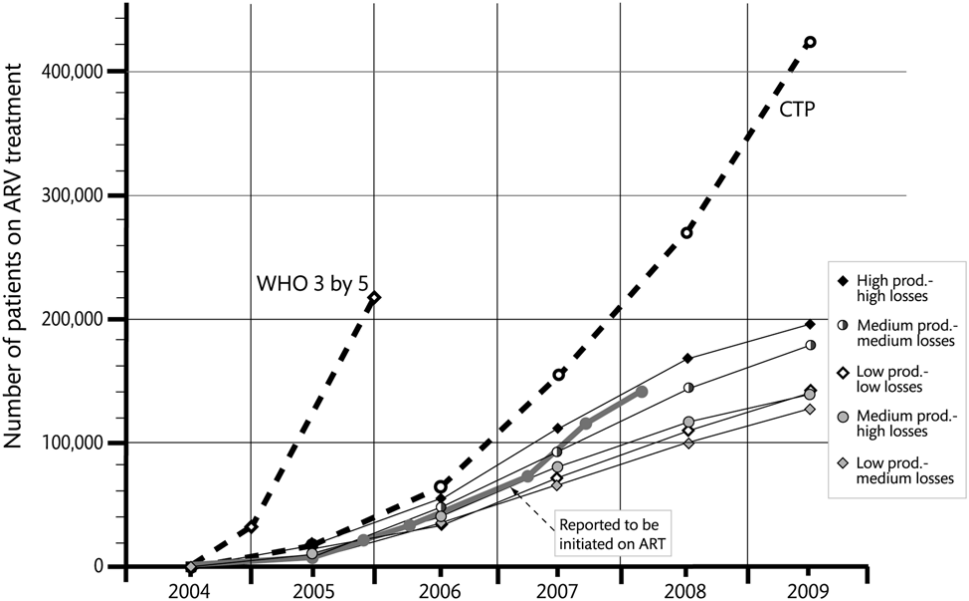
ART output. The number of patients on ART in Tanzania according to end of year numbers of WHO's “3 by 5” initiative and mid-year numbers of CTP and five scenarios. Finally the reported number of patients initiated on ART as the thick grey line running up till the end of February 2008.

Finally, we calculated the ART output and the number of life years that could be saved for five different scenarios with three different levels of clinical staff productivity and patient losses. The first calculations were made in the beginning of 2006 and later compared with the actual reported number of patients *initiated on ART* at the end of February 2008 as well as with the output planned in the CTP (figure 2). The details of this process are described below.

### The Tanzanian Care and Treatment Plan (CTP)

By mid-2009, the CTP aims to have 420,000 HIV positive individuals with CD4 counts below 200/ml on ART, and to monitor 1.3 million patients not yet in need of treatment. It assumes an annual loss of patients on ART of only 1%, equal to the age-specific mortality of the general population. To enable this scale-up, the number of accredited Care and Treatment Centres (CTCs) in mainland Tanzania (excluding Zanzibar) must increase from 20 to 240 centres in less than 4 years (table 1).

**Table 1:** Planned number of patients on ART in Tanzania and in brackets the planned number of health care facilities accredited at the end of each fiscal year (F_y_) according to the CTP.

Type of hospital	2004/5	2005/6	2006/7	2007/8	2008/9
Referral & Selected Regional	10,000 (10)	20,000 (10)	30,000 (10)	40,000 (10)	50,000 (10)
Remaining Regional	1,800 (3)	9,600 (13)	17,400 (13)	25,200 (13)	30,000 (13)
District Voluntary & Private	4,200 (7)	34,800 (51)	102,800 (113)	207,600 (175)	340,800 (217)
Total (**F_y_**)	16,000 (20)	64,400 (74)	150,000 (136)	272,800 (198)	420,800 (240)

According to the plan, CTCs should be located at hospitals and run by treatment teams of 18 staff members including 7 counsellors. The staff requirements for the CTP were derived from estimating the need for consultation time per patient for the first and subsequent years on ART for each staff category, including clinicians, counsellors, pharmacists and laboratory personnel (table 2). In all around 9,000 full time equivalents of clinical staff need to be recruited for treatment and monitoring, 1200 of whom should be clinicians (table 2). Clinicians are divided into *prescribing* (medical officers with 6 years of training) and *evaluating* clinicians (clinical officers with 3 years of training or assistant medical officers with 5 years of training). The average consultation time required per first-year patient on ART, is estimated to 4 hours (2 visits à 30 minutes and 9 visits à 20 minutes) and after the first year, 1.3 hours (4 visits à 20 minutes) of clinician care per year and patient. Thus, three times more times is assumed to be required for first year than for post-first year patients As staff is estimated to work 800 hours with patients per year this translates into 200 first-year patients (newly initiated on ART) and 600 post-first-year patients annually for every clinician [11]

**Table 2:** Number of patients in thousands on ART and CD4 monitoring by end of fiscal year in Tanzania; staff requirements and budget for each fiscal year according to the CTP.

	2004/5	2006/7	2008/9
**Number of patients in '000s**			
**Patients on ART**	**16**	**151**	**423**
New patients on ART	16	86	149
Eligible patients	160	160	160
Coverage (% of eligible patients)	10%	54%	93%
**On CD4 monitoring**	**49**	**454**	**1269**
New patients on CD4 monitoring	49	258	445
**Total staff for ART and CD4 monitoring**	**432**	**3481**	**8802**
Total staff for ART	228	1861	4804
Clinicians	80	539	1202
Nurses/counsellors	105	971	2690
Total staff for CD4 monitoring	204	1620	3998
Clinicians	76	655	1605
Nurses/counsellors	95	719	1781
**Annual budget (million USD)**	**26**	**98**	**207**
Antiretroviral drugs+laboratory tests	11	66	147
Salaries*	1	9	24

* The external input into salary costs.

### Situation analysis and resulting assumptions and estimates

The annual number of HIV-infected Tanzanians in immediate need of ART was estimated to 160,000 new individuals assuming stable incidence rates (as those currently observed) [14]. By November 2005, 19,600 patients had started ART [2], by March 2006, 35,000, by September 2007, 117,000 by the end December 2007, 136,000 [15] and by the end of February 2008, 143,000 had been initiated on ART and 277,000 were being monitored (pc NACP), indicating that the rate of scaling-up access is slower than predicted by the CTP (figure 2). However, no data is yet available, on the degree of under- or duplicate reporting, or on the number of patients lost to follow-up or death. Thus, these figures need to be estimated based on existing sources of information.

#### Estimation of staff availability

In our model we assumed that the availability of staff would be the main limiting factor of ART scale-up and that clinicians would be *the main limiting staff factor* for ART scale-up. HIV patients do not need to see a medical doctor, but they need to meet clinical staff trained to assess history, signs and symptoms and who can prescribe and evaluate the effect of ARV drugs. The IMF-induced hiring freeze of government health staff in 1993 has resulted in an old workforce where more than 15% are 50 years or older. The total number of health workers, of which 65% work in government services, 22% in private-not-for-profit and 14% in private-for-profit institutions, has declined from 67,000 in 1995 to 54,000 in 2002 and has been estimated to fall even further to 49,000 by 2015 [19]. In 2001/2, only 60% of health staff positions deemed necessary by the Ministry of Health were filled, and has since declined even further mainly due to increased demand through a rapid population increase (pc Dr Njau MoH). The hiring freeze was lifted in 2004, but due to a continued high attrition rate and the long lag-time to expand health worker training, only a limited increase of government staff could be expected up to 2009 since back-migration or large numbers of health workers returning from private care are unlikely.

According to the national plan, mainly hospitals should be accredited CTCs for ART provision, but since there are only about 190 hospitals suitable for ART provision, most of them in urban areas, an expansion to primary health care level in rural areas with even fewer staff per facility will be necessary to reach high ART coverage. Thus, in contrast to the CTP, we took into consideration that less staff per facility would be available at the primary health care level after CTCs have been established at all available hospitals.

Also, since half of the 1.4 million HIV infected Tanzanians live in rural areas [20], [21], some patients must receive treatment at primary health care level. Although less than 2% of the patients will be treated outside hospitals during 2004–2009 we still included this in our scenarios since it affects the outcome for the last two years when the expansion to the primary care level has to take place.

Considering the shortage of medical officers we assumed that both prescription and evaluation will have to be done by any clinical staff available and categorized both prescribing and evaluating clinicians as “clinicians”. Using the 2001/2 Ministry of Health figures of existing clinicians, we calculated the average number of clinicians per type of health facility and further assumed, in line with previous estimates [22], that the average number at each type of facility, from health centres to regional hospitals, would remain the same from 2002 throughout 2009 in all scenarios, but that service provision would be scaled-up at the pace outlined in the CTP (table 1).

#### Estimation of staff performance

Few clinicians spend all their HIV working time on ART. Other activities include voluntary counselling and testing (VCT), condom distribution, education in schools, prevention of mother-child transmission (PMTCT), treatment of STIs, palliative care, monitoring of immune status and treatment of opportunistic infections in patients not yet qualifying for ART.

In Rufiji district, with an adult HIV prevalence in 2003 of 7%, similar to the country average, sexually transmitted infections (STIs) including HIV/AIDS constituted 22% of the burden of disease (BoD) [16]. It has also been estimated that 35% of all health staff time in Tanzania should be invested in HIV/AIDS interventions to reach the Millennium Development Goals (MDGs) of 40% ART coverage by 2015 [17], [18].

*Thus, we assumed that that a maximum of 20% of clinicians' patient time would be devoted to ART. A higher proportion would affect both other HIV activites and other priority health interventions as assessed by WHO's Commission on Macroeconomics and Health* [*18*]

The CTP expects prescribing and evaluating clinicians to work 800 hours/year and to treat 200 first-year patients (newly initiated on ART) and 600 post- 1^st^-year patients per year [11]. However Kurowski et al observed that clinicians at Tanzanian government hospitals and health centres spent an average of 40% of their working hours on patient care, i.e. 640 hours/year [19], i.e. 160 hours less per year than the 800 hours/year assumed in the CTP plan.

*Thus, we used 640 hours/year as our low-productivity estimate, while a 40% productivity increase corresponding to 900 working hours per year per clinician was considered a medium productivity scenario and a doubling of the by Kurowski et al observed productivity would be considered high productivity, in our scenario analyses*

#### Estimation of mortality rate and loss of patients on ART

The 1% mortality rate assumed by the CTP is most likely an underestimation since data from seven ART programmmes providing ARVs free of charge showed an average mortality of 11% after 6 months [3] while a first-year mortality of 14% was reported from South Africa [5]. The high initial mortality may possibly be explained by data both from Tanzania (pc Dr Kaushuk, Hindu Mandal Hospital, Dar es Salaam) and other low-income countries [23] indicating that a large part of all diagnosed HIV patients seek care very late with CD4 counts below 50/ml. However, a study from Uganda found a very high first-year mortality of 18% even among patients starting ART with CD4s counts above 50/ml [4]. A study from Botswana of 410 patients on ART with a median follow-up of 44 weeks, found that 24% had been lost to follow-up or death. The reported first-year survival rate was 79% (95% CI 74–81%) [6]. A recently published study from the national programme in Malawi confirmed the findings of a high first year mortality estimated to 18.5% [29]. *Hence, we assumed that patients newly initiated on ART would have an average first-year mortality of 15% in addition to a basic age-specific mortality of 1%* [24].

Hospital-based fee-for-service programmes in Uganda and Malawi show one-year patient losses of 56% and 59%, respectively, mainly due to lack of money among patients and irregular drug supply at health centres [25], [26]. Small well-controlled programs in Malawi and Kenya providing ARVs drugs free of charge, report annual first-year losses of 10%–22%, increasing to 30% in Kenya after the second year of ART [27], [28]. A meta-analysis shows a program retention rate of 40% among ARV patients after 2 years in sub-Saharan Africa [23]. The recent above mentioned study of the Malawi national programme showed patient losses of around 10% during the first year [29]. Apart from the Malawi study we have not found any other published data on patient losses from scaled-up national ART programs in low-income countries in sub-Saharan Africa. *In this study, we assumed both a low loss scenario of 10%, a medium-loss scenario of 20% and a high loss-scenario of 40%*.

### The conceptual framework

Our situation analysis resulted in a conceptual framework with four major determinants of ART output in Tanzania (figure 1):

1. *staff availability and productivity,*
2. *patient-related factors including adherence, stigma, education level, socio-economic situation and family support,*
3. *management and logistics and,*
4. *financial resources, drugs and laboratory supplies.*

and with two main factors limiting service provision during the current five-year planning period 2004–2009: 1) the availability and productivity of clinicians and 2) retention of patients in the treatment programmes. We assumed an average duration of 9 years from infection to AIDS [30]. Although this figure has lately been adjusted after new research findings [31] we still retained it as plans had been based on it and we had to have comparable figures. Since most patients are initiated on ART when they already have clinical signs of AIDS, monitoring of immune status among those in WHO stage I and II will be of limited importance. In order to reduce the number of uncertain input variables in the formula, we assumed that donors will provide sufficient management support, financial resources, drugs and laboratory supplies during this *initial* five-year period (not saying that current levels will be sufficient in a future scaled-up context) and that it will be possible to use existing staff for counselling and supportive functions given the relatively small number of patients currently enrolled. We also judged that stigma and low awareness would not affect the demand for services during the implementation period 2004–2009 since attendance of VCT has increased rapidly during the last years and since many HIV positive women are diagnosed at antenatal care clinics and make up over 60% of all patients on ART in Tanzania (pc NACP).) We also assumed that patient related factors listed above will be largely captured in our *patient loss* estimates.

### Estimation of the number of patients on ART each year

**Figure 3:**
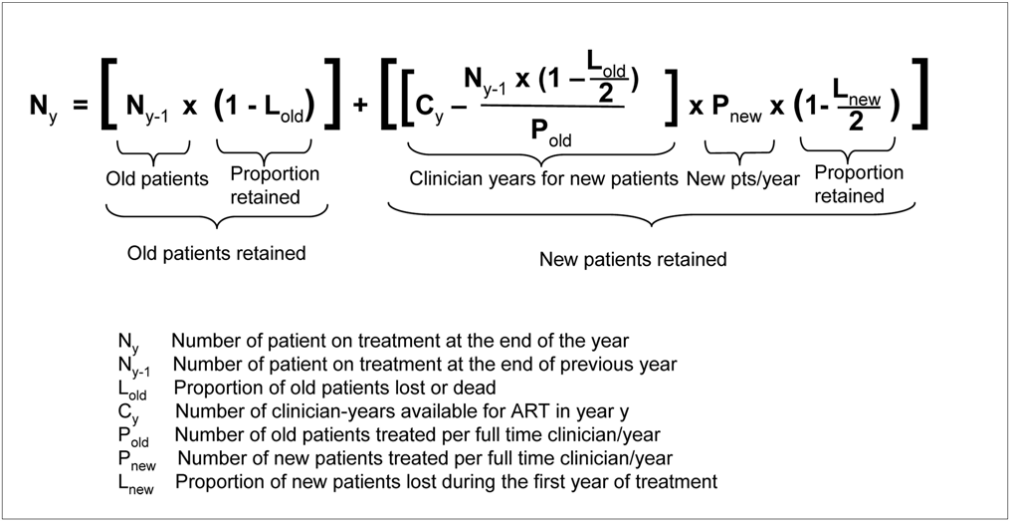
Formula for the calculation of ART output.

We constructed a simple dynamic model to estimate the number of new and post-first-year-patients on ART at the end of each year based on annual availability and productivity of clinicians, and, on the proportion of patients lost to follow-up or death,. The variables in this formula (figure 3) include: *first year mortality, age-specific mortality*, *other patient losses (loss to follow-up), average number of clinicians per facility type, time devoted to HAART, new patients/year, old (post-first-year) patients/year(clinician productivity).* The input values of each variable can easily be changed by entering new data into a spread sheet. For our scenarios we only varied *patient losses, new patients/year* and *old patients/year( clinician productivity)*, but kept the other variables constant.

#### “Old” (post-first-year) patients retained and under continued treatment

In the formula (figure 3) the number of old patients retained is determined by the number of patients on treatment at the end of the previous year **(N_y-1_)** and the loss of patients until the end of the year **(L_old_)**. Clinician-time available to care for old patients **(N_y-1_×(1-L_old_/2)/P_old_)** is determined by the number of these post-first-year patients retained in the ART program at the beginning of each year **(N_y-1_)**, multiplied with the proportion of old patients retained after an average of half a year (assuming that patient loss-to-follow-up or death is equal throughout the year and thus the average time in the program for a patient that drops out for one reason or the other is 6 months) **(1-L_old_/2)**, divided by clinician productivity measured as the number of old patients a full-time clinician cares for during one year **(P_old_)**. We did not take re-entry of lost patients into account and consistently use the term “old” patients for all that have been on ART for more than 1 year.

#### Total clinician-time (C_y_) available for ART care

Total clinician-time **(C_y_)** available is calculated as the number of accredited CTCs available to offer ART services a particular year y **(F_y_)** (table 1), multiplied with the average number of clinicians at the health facilities **(C_ave_)**(table 3) multiplied with the proportion of time clinicians devote to ART of their total working time **(T_ART_)** (table 4).

**Table 3:** Actual and authorized number of clinicians at different types of health facilities in Tanzania in the fiscal year 2001/2.

Type of health facility	Number of facili-ties	Actual mean number per facility (C_ave_)	Authorized mean number per facility	Actual number per type *	Authorized number per type **
Referral Hospitals	4	39	58	157	232
Regional Hospitals***	19	15	8	287	152
District Hospitals	184	11	21	2053	3864
Health centers	397	3	4	1016	1588
TOTAL	604	5.8	9.7	3513	5836

* Ministry of Health/Civil Service Department Staffing levels for health facilities/institutions. Dar es Salaam 1999.

** Ministry of Health, Census of Human Resources for Health for 2001/2.

*** Includes one military hospital.

**Table 4:** Input estimates for productivity and losses and outcomes obtained mid-year 2009 for the five scenarios and the CTP based on the formula.

PLAN AND SCENARIOS		*CTP*	Low prod-low losses	Low prod-medium losses	Medium prod-medium losses	Medium prod-high losses	High prod- high losses
**INPUT**							
Time for ART - % of full time	**T**_ART_	*100%*	20%	20%	20%	20%	20%
New patients/clinician-year 1^st^ year	**P**_new_	*200*	160	160	225	225	320
Old patients/clinician-year after 1^st^ year	**P**_old_	*600*	480	480	675	675	960
Lost patients first year*	**L**_new_	*1%*	26%	36%	36%	56%	56%
Lost patients after 1^st^ year**	**L**_old_	*1%*	11%	21%	21%	41%	41%
**OUTPUT**							
Coverage in 2009		*93%*	25%	27%	38%	41%	58%
No on treatment in 2009		*420,000*	140,000	125,000	176,000	138,000	251,000
LYs saved 2004–2009		*710,000*	287,000	254,000	358,000	274,000	509,000
AIDS deaths without access to ART 2004–2009***		*380,000*	642,000	639,000	574,000	579,000	485,000

* includes age-specific mortality of 1%+losses to follow-up and non-adherence+1^st^ year AIDS mortality.

** includes age-specific mortality of 1%+losses to follow-up and non-adherence.

*** total number of AIDS deaths minus the sum of the number of new patients per year 2004–2009.

#### “New” (first-year) patients recruited for ART

The number of new patients that can be recruited depends on 1) the productivity of clinicians 2) the consultation time clinicians need for “old” (i.e. post-first-year) and how much time is left over for the new patients after the “old” patients have been treated 3) how much time is needed to initiate a new patient on ART (i.e. 4 hours/year according to the CTP). The number of new patients retained at the end of each calendar year is thus determined by: 1) the amount of clinician-time available for recruitment of new patients multiplied with 2) the number of new patients per clinician year **(P_new_)**, multiplied with the proportion of new patients remaining after on average half a year on treatment **(1-L_new_/2)**.

We calculated the number of patients per clinician per year, both for the first year of treatment and the following years. Total ART output was defined as the sum of outputs from all accredited ART facilities that year. ART coverage was defined as the annual number of new patients initiated on ART divided by the estimated number of 160,000 eligible new HIV-infected individuals that annually need ART.

### The scenarios

Based on our situation analysis, the resulting assumptions and the above-defined formula, we created five scenarios with varying levels of *staff productivity and patient loss*, to demonstrate the effect on the number patients that could access ART (ART output) in Tanzania.

Using results from Kurowski et al [19] who observed 640 working hours/year/clinician, divided by 4 hours total clinician consultation time per year per new ART patient and 1.3 hours per patient for each subsequent year on ART respectively as assumed for the CTP, we outlined a *low productivity scenario* of 160 (640/4) first-year patients (P_new_) or 480 (640/1.3) post-first-year patients (P_old_) per working year per clinician. If productivity is raised by 40%, *defined as medium productivity* i.e. to 900 working hours per year per clinician, the input per year per clinician is 225 first-year patients or 675 post- first-year patients. If productivity is doubled, defined as *high productivity*, 320 first-year patients or 960 post-first-year patients would be treated per clinician per year.

*Low loss* is defined by age-specific mortality 1%+first year mortality 15% and patient losses due to other causes of 10%. *Medium loss* is defined by age-specific mortality 1%+first year mortality 15% and patient losses due to other causes of 20%. *High loss* is defined by age-specific mortality 1%+first year mortality 15% and patient losses due to other causes of 40% (table 4).

#### Number of AIDS deaths and life years saved

**Figure 4:**
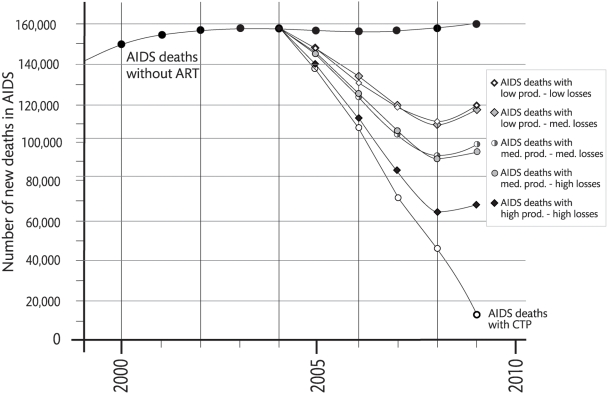
AIDS deaths. Estimated number of AIDS deaths without ART from 1999 to 2009 and with ART according to the five scenarios and to the CTP from 2004 to 2009.

We also estimated the effect both of the CTP and the five scenarios on the number of projected AIDS deaths (figure 4). The number of new AIDS deaths were generated by entering the Estimation and Projection Package (EPP) data [32] from the sentinel surveillance sites in Tanzania of the latest surveillance round 2005/6 [10] into the Spectrum model [9]. The number of patients initiated on ART (new patients) according to the CTP and the four scenarios were then deducted from the projected new AIDS deaths without ART to calculate the effect of ART implementation. Life-years (LY) saved were defined as the sum of half of the patient-years saved for 1^st^ year-patients, i.e. the average of six months saved per year, assuming an equal enrolment rate across the year, and patient-years saved for old patients during the same period.

We also made a sensitivity analysis for *patient losses*, *number of clinicians*, and *first- year mortality*.

## Results

Figure 2 displays the maximum number of HIV positive individuals that could be treated with ART (ART output) in Tanzania in five different scenarios with the current human resource capacity and planned pace of accreditation of new treatment centres, varying staff productivity and patient loss. Our estimations are displayed together with the initial WHO plan and national Care and Treatment Plan (CTP) targets for ART scale-up in Tanzania during 2004–2009 for comparison. Input and outcome values for the five scenarios, the WHO plan and CTP are shown in table 4.

The productivity and patient loss assumptions have been defined in detail in the Methods section. In all scenarios a first-year mortality of 15% and an age-specific mortality of 1% was assumed, but the loss due to other causes, such as loss-to follow-up or drop-out from treatment, varied from 10% (low), to 20% (medium) up to 40% (high) loss.

The first scenario; “low productivity-low loss”, assumes that productivity remains at the level observed in 2003 [19] with a low rate of patient loss. If this scenario would be implemented, 140,000 would be on HIV treatment by mid-2009, reaching an ART coverage of 25% (table 4). Continued low productivity means clinical staff would devote a large proportion of their time to those already on ART (old patients) (100,000) and enrollment of new patients during 2009 would remain low (40,000). The number of life-years (LYs) saved (287,000), during 2004–2009 would mainly reflect prolonged survival of patients already enrolled. Around 80% of HIV-positive individuals who progress to AIDS will die from the disease during the same planning period, without having been initiated on ART (figure 4).

The second scenario; “low productivity-medium loss”, shows the outcome of continued low productivity but with a slightly higher level of patient loss. This would lead to a slightly higher ART coverage of 27% in 2009, as the higher loss of previously enrolled patients would enable more new patients to be enrolled but the number of LYs saved would be lower at 254,000 (Table 4). The proportion of all who develop AIDS and die without having been initiated on ART would remain as high as 80% (figure 4).

If productivity is increased by 40% as in our third scenario, the “medium productivity-medium loss”, ART coverage would increase to 38% in 2009. The number of new HIV patients recruited for ART in 2009 could increase to 61,000 while 115,000 previously enrolled patients could be maintained on treatment (not shown in table). The number of LYs saved would be a little over 350,000.

If we use the productivity figures of the CTP of 200 and 600 patients per year for first and post-first year patients respectively and assume medium losses, the results are similar with a coverage of 38% and a number of LYs saved of 320.000

If the fourth “medium productivity-high loss” scenario would materialise, which assumes a 40% patient loss due to other causes than death, ART coverage would increase to 41% in line with the discussion above. The number of LYs saved would decrease to 274,000 (table 4).

The fifth scenario “high productivity-high loss” assumes that clinicians become twice as productive, i.e. that they either double their working time or double the number of patients seen without increasing the number of hours. This scenario would result in 251,000 of those in need having access to ART corresponding to a 58% ART coverage in 2009, 500,000 LYs would have been saved and an equal number of people dying without having had access to ART (table 4).

Assuming figures on first year mortality of 18.5% and patient losses of 10% observed in Malawi [29] and still that three times more hours are put into treating first year patients than into treatment of patients after the first year and an output of the reported number of patients initiated on ART of 143.000 in February 2008 the average staff production would be 210 first year and 630 post-first year patients per year. This corresponds well with our medium productivity scenarios but with a lower loss rate.

Figure 4 shows the proportion of new patients that will get access to ARV according to the five scenarios out of the 160,000 HIV infected estimated to develop AIDS every year in Tanzania. The numbers in the figure are derived from our scenario calculations and based on the assumptions that resources first will be devoted to previously enrolled patients and that the current productivity level remain constant.

Thus, Table 4 and Figure 4 shows that if the CTP was implemented, 420,000 patients would be on ART and 710,000 LYs will be saved by 2009. Meanwhile, another 380,000 will die from AIDS during 2004–09 without having accessed ART (table 4)

If the medium productivity-medium loss or the medium productivity–high loss scenarios were implemented, the number of AIDS deaths would be reduced by 40% from 160.000 to around 95.000. Thus, most infected people would still die without ever having had access to ART.

A sensitivity analysis for the parameters *patient losses* (losses due to other causes such as loss to follow-up), *first year mortality* and *the average number of clinicians per facility type* was performed. It shows that a 1% change in the *average number of clinicians* directly translated into a corresponding 1% change in ART output irrespective of scenario. A 1% change in *patient losses* resulted in 0.1% change in ART output in the low productivity-low loss-scenario, a 0.2% change in medium loss scenarios and a 0.5% change in the high loss scenarios. A 1% change in *first year mortality* resulted in a very small change of service output, less than or equal to 0.1% in all five scenarios.

## Discussion

We aim to estimate the capacity for ART provision in Tanzania. Using a simple user-friendly model we are able to demonstrate that the current plans of scaled-up access to ART in Tanzania were never feasible. Reality also shows that the implementation of ART is progressing at a slower pace than foreseen in the national Care and Treatment Plan (CTP). This papers shows both that the major reasons why targets have not been reached, are that the current plans assume a constant availability of sufficient staff and that the loss of patients on ART has not been taken into account and that it is possible to estimate achievable targets if a limiting staff factor can be identified.

Our estimations suggest that around 175,000 patients will be on ART in Tanzania by mid 2009, which is 40% of the target in the CTP (figure 2). The reported number of 143,000 initiated on ART by end February 2008 may be either an underestimation since many smaller sites did not sent their reports (pc NACP), or an overestimation since re-entry at the same or at a different CTC is not taken into account. Moreover, the reported number available is an accumulation of patients ever initiated on ARV without consideration of patients lost to follow-up or death. The exact actual number of patients on treatment end February 2008 is thus not known, but the magnitude is likely to be around the number reported to be *initiated on ART*. This number largely coincides with what our medium productivity-medium-loss scenario predicted two years earlier. This indicates that estimating more achievable targets based on our assumptions is feasible.

In our model we see the ART provision as a result of staff productivity and patient losses. We also assumed that possible weaknesses in management would not be limiting during the implementation period. The actual production may have been even smaller if for example poor management capacity limits scale-up. In the model we did not include that, but estimated what maximally could be achieved by the existing system under three main assumptions (figure 1):

- Clinicians (number and productivity) is the main limiting staff factor. We estimated they would spend 20% of their working time on ART
- Patient losses (deaths and losses to follow-up) constitute a measure that also captures the sum of all other patient factors including stigma, transport etc.
- The other factors, logistics, management and funding are not limiting during the implementation period

If all these assumptions are in agreement with the actual findings in a country our estimations should be valid. Variations in the number of clinicians and staff productivity are captured by the model as are variations in patient factors through losses. The time devoted to ART is part of the function.

Setting achievable targets is important for planning including estimations of resource needs. Even if the CTP is implemented according to plan with 420,000 on ART in 2009 (fig. 2), this implies 380,000 potentially avoidable AIDS deaths during 2004–2009 – a figure which, however, for our scenarios is considerably higher. Our predictions suggest that the number of projected AIDS deaths will be reduced by about 40% - from 160,000 to about 95,000 if implementation follows the medium productivity scenarios (figure 4) and that potentially avoidable AIDS deaths instead will be around 575.000 (table 4).

Our analysis indicates that the main limitation for ART scale-up is the low number and low productivity of clinical staff. The rapid increase in ART initiations after March 2007 might indicate that losses are high, making it possible to initiate more new patients on treatment. However, patient loss does not affect output as much since losses of previously enrolled patients on ART allows for recruitment of new patients (table 4), but the effects of patient losses are larger for the high loss scenarios (see sensitivity analysis under results).

The shortage of staff limits both recruitment of new patients for ART and further reduction of the number of AIDS deaths. The scarcity of trained clinical staff will be further accentuated during the last two years of the planning period by the expansion of ART services from hospitals to lower level health centers with much fewer clinical staff. An increase in the number of clinical staff available and/or an increase in productivity of existing clinical staff would be needed to counter the tendency of increase in the number of avoidable deaths due to AIDS shown in the scenario curves after 2008 (figure 4). Some reductions might also be achieved through additional task shifting. However, current staffing levels are <60% of the levels authorized by the government [22]. The employment freeze from 1993 resulted in a declining cadre of qualified health personnel [21] that is now over-aged with high attrition. The freeze was lifted in 2004 and the outputs from training institutions have increased for medical officers, assistant medical officers and clinical officers [33], but many of these were recruited from within the workforce and as recruitment of new staff is a slow process [34] it will take several years before any real increase in staff numbers can be observed. The annual population growth of around 800.000 in Tanzania also leads to a rapid increase in demand for health care. In fact – and maybe mainly because of this - the staffing levels seem to have continued to decrease (pc Dr Njau, MoH).

Some of the assumptions may also not be correct. Accurate estimates for planning of ART require evidence on staff productivity and patient losses in scaled-up program in highly affected low-income countries. Such evidence is so far only available for some parameters from one large national programmes in sub-Saharan Africa [29] and therefore the staff input values used in our scenarios are based on findings from smaller treatment program and studies. Our staff productivity figures are based on a small sub-study in Tanzania [19].

The CTP targets are more achievable than those set by the WHO, but our study shows that also CTP underestimated existing constraints for ART in the Tanzanian health system. A possible increase in the number of qualified health staff for ART will also compete with other important health care needs including other priority HIV interventions, such as male circumcision [35], [36], PMTCT or HIV research projects. But, to reach higher coverage it is necessary that HIV incidence is reduced [37].

The monitoring of patients not yet eligible for ART (CD4 counts >200) would, according to the CTP, require almost as many staff as is required to treat patients in acute need of ART and 30% more clinicians in 2009 (table 2). Thus, if we had included the CD4 monitoring part of the CTP in our scenarios the estimated ART coverage would have been even lower. An indirect conclusion of our study is that ART initiation in Tanzania must be based on clinical staging rather than on resource-demanding CD4 monitoring using the so called public health approach, also recommended by WHO for similar contexts [29], [38], [39].

Our assumption that clinicians rather than nurses, pharmacists and counselors, will be the main *limiting facto*r for ART scale-up may be either overly optimistic or overly pessimistic given the lack of existing evidence of clinician-time requirements for ART patients, but it does not restrict the use of the model since *it can* be applied to any type of limiting staff category.

Our model estimates what maximally could be achieved by the existing system under the assumptions outlined in the Methods section. Here we see patient loss as a summary measure that captures the net effect of all patient related factors including stigma, socio-economic capacity and transportation costs. These losses could be seen as a result both of patient factors and health system/management factors, mainly access to and quality of care. The loss of patients would be less if the quality services and the availability of drugs improved. Experiences from other sectors and from STI-scale-up, indicate that there is a risk of that scaling-up leads to reduced system effectiveness due to limited management and logistics capacity [40, Hanson unpublished]. On the other hand, ART programs could be strengthened through cooperation with strong NGOs as in Uganda [41], or through public/private mix - a feasible approach at least in middle-income countries like Botswana [42]. Thus, alternative providers may become important if they are more productive than the public service staff. This increased productivity then has to put into the model.

Although our simple model does not explicitly capture the function of the system and the effects of scaling up, it has the advantages that it could be applied to any limiting staff category, to any country, is based on data that is routinely collected or that could be collected relatively easily through surveys, and contributes to the understanding of what factors are critical for ART provision.

In conclusion, the agreement in magnitude between scenario and reality two years later suggests that it is feasible to outline achievable scale-up plans also in low resource settings like Tanzania. The model demands identification of limitimg factors and gives the magnitude of what can be produced under each scenario. As improved input data become available the range of scenarios can be narrowed and the estimations improved. This would make it possible to further improve planning and resource allocation.
